# Automated screening devices for vision screening in preschool children: A comparison of the PlusoptiX S12C photoscreener and retinomax K+3 autorefractor

**DOI:** 10.3389/fopht.2022.1049622

**Published:** 2022-12-22

**Authors:** Stephen C. Hunter, Donny W. Suh, Iliana Molina, Jennifer Espinoza

**Affiliations:** ^1^ University of California Riverside School of Medicine, Riverside, CA, United States; ^2^ Department of Ophthalmology, Gavin Herbert Eye Institute, University of California Irvine School of Medicine, Irvine, CA, United States

**Keywords:** vision screening, children, amblyopia, refractive error, amblyopia risk factor, photoscreener, autorefractor

## Abstract

**Introduction:**

Automated vision screening devices such as photoscreeners and autorefractors have been used to accurately identify amblyopia, refractive amblyopia risk factors (ARFs), and refractive error in young children; however, there is conflicting data about the effectiveness of different screening devices. We compared the performance of two commercially available screening devices in preschool children.

**Methods:**

Children aged 3 to 5 years attending 5 preschools in Anaheim Elementary School District were screened with the PlusoptiX S12C photoscreener using ROC 3 referral criteria and Retinomax K+3 autorefractor in March 2022. Screened children were offered free cycloplegic eye examinations performed by optometrists on the UCI EyeMobile for Children mobile clinic. Children were evaluated for the presence of refractive ARFs using 2021 American Association for Pediatric Ophthalmology and Strabismus age-based referral criteria guidelines for instrument-based screening.

**Results:**

A total of 158 children were screened and 79 children received cycloplegic examinations. At least one refractive ARF was found in 20% of examined children, corresponding to a sensitivity/specificity/positive predictive value (PPV)/negative predictive value (NPV) of 94%/89%/68%/98% for the PlusoptiX and 100%/65%/42%/100% for the Retinomax.

**Discussion:**

In detecting refractive ARFs, the PlusoptiX was found to have a higher specificity and PPV while the Retinomax had a higher sensitivity and NPV. While both devices demonstrated a high sensitivity and NPV, we found that the PlusoptiX performed better overall as a screening device for our program as the Retinomax referred too many children.

## 1 Introduction

Periodic vision screenings are especially important for young children, as undetected visual disorders such as amblyopia and refractive error may hinder a child’s social and cognitive development or lead to permanent vision loss (1–3). The early detection of visual impairment is critical as it facilitates early intervention, which is associated with improved outcomes and increased treatment cost-effectiveness in children with amblyopia (4–6). The US Preventive Services Task Force currently recommends at least one vision screening for all children aged 3 to 5 years, which is consistent with recommendations from the American Academy of Ophthalmology (7, 8).

Advancements in technology have changed the landscape of pediatric vision screening over the past 3 decades. Although they can be costly, automated screening devices such as photoscreeners and autorefractors offer advantages over traditional methods using visual acuity in that they are faster and may be used to screen preverbal children. They are accurate, reliable, and have been validated, which has led to their use by pediatricians, school nurses, and community vision screening programs (6, 9–11). Despite their widespread use and proposed benefits, there is conflicting data about the effectiveness of different automated screening devices (12).

The purpose of this study is to compare the accuracy of the PlusoptiX S12C photoscreener and Retinomax K+3 autorefractor in determining refractive amblyopia risk factors (ARFs) in preschool children aged 3 – 5 years within a community vision screening/treatment program.

## 2 Materials and methods

In March 2022, children 3 – 5 years of age attending 5 preschools in Anaheim Elementary School District were screened at their preschools. Students who were absent on the dates screening took place and those who did not obtain parental consent were excluded from screening.

The University of California Irvine (UCI) EyeMobile for Children is a community vision screening program for preschoolers modeled after the University of California San Diego EyeMobile for Children program. The program screens children attending preschools in Orange County and provides comprehensive eye examinations, glasses prescription, and referral, if necessary, to children who fail screening. Examinations are performed on a mobile eye clinic that visits the child’s preschool at a later date. All eye care services are provided at no cost to families.

Screenings were conducted by two trained screeners. One screener performed screenings using the Retinomax K+3 autorefractor (Righton, Tokyo, Japan) and the other screener performed screenings using the PlusoptiX S12C mobile photoscreener (PlusoptiX GmbH, Nuremberg, Germany). The PlusoptiX includes five receiver operating characteristic (ROC) modes with variable referral criteria based on specificity and sensitivity levels set by the manufacturer. Referral criteria for the PlusoptiX were based on ROC 3, corresponding to 85% sensitivity and 90% specificity per the manufacturer. Children were referred by the PlusoptiX if the device detected any of the following: hyperopia ≥ +3.50D, myopia ≥ -3.00D, astigmatism ≥ 1.50D, anisometropia ≥ 1.00D. Referral criteria for the Retinomax were: hyperopia ≥ +1.75D, myopia ≥ -3.25D, and astigmatism ≥ 1.50D, anisometropia ≥ 1.50D. Children with inconclusive screening results were considered to be referred by the device. All children were screened by both devices in the same setting with the order of testing randomized. Screenings were conducted in classrooms where lighting conditions were not consistent. If screening result was not obtained on the first attempt, at least two additional attempts were made.

All parents of screened children were provided with the result of the screening examination and given a consent form for a free comprehensive eye examination. Examinations were performed by two pediatric optometrists and consisted of near and distance visual acuity using HOTV, cover testing, autorefraction before and after cycloplegia, cycloplegic and non-cycloplegic refraction (“wet” and “dry” retinoscopy), anterior segment examination, and fundus examination. Cycloplegia was attained after topical installation of cyclopentolate 1% drops. All examinations were completed within 3 months of the initial vision screening.

Examination records were retrospectively reviewed. Examination data from children who did not receive cycloplegia were excluded from analysis. Hyperopia and myopia were calculated as spherical equivalent (SE). SE was calculated as the sum of the spherical plus half the cylindrical error. Amblyopia was defined as unilateral if there was a ≥ 2-line difference in the best corrected visual acuity (BCVA) between the eyes with the presence of an ARF, and bilateral if the BCVA in each eye was < 20/50 for children < 4 years old, < 20/40 for children 4 to 5 years old, and < 20/30 for children older than 5 years with the presence of an ARF. Presence of ARFs was determined using 2021 American Association of Pediatric Ophthalmology and Strabismus (AAPOS) age-based referral criteria guidelines for instrument-based screening (13). Referral criteria for all children include anisometropia > 1.25D and hyperopia > 4.0D; for children aged 31-48 months, astigmatism > 3.0D and myopia < -3.0D were used as thresholds; for children > 49 months of age, astigmatism > 1.75D and myopia < -2D were used instead. Criteria were applied to cycloplegic retinoscopy data.

## 3 Results

158 children attending 5 preschools in Anaheim Elementary School District were screened in March 2022. The PlusoptiX referred 46 children (29%), 9 of which were classified as “unable.” The Retinomax referred 82 children (52%) with no inconclusive screening results. Of all screened children, 82 (52%) obtained parental consent and were examined. The remaining 76 screened children were not examined either due to parental decline, lack of consent, or because they did not show up for their appointment. Among the children who received examinations, 3 children (4%) did not need glasses per the optometrist’s clinical judgement based on VA and dry retinoscopy data and were not given cycloplegic examinations. The 3 children who did not receive cycloplegic examinations were not referred by either device upon screening.

A total of 79 children received comprehensive cycloplegic examinations. The age range of examined children was 40 – 65 months with a mean age of 4.7 years. Right eye refractive error results for children given a full cycloplegic examination are presented in **Table 1**. Hyperopia was the most common refractive error, found in 66% of examined children with a mean of +1.37D (range 0.5 – 5.75). Myopia was found in 8% of children with a mean of -0.79D (range -1.25 – -0.50), and 26% were found to be emmetropic with a mean of +0.14D. Astigmatism was found in 44% of examined children with a mean cylinder power of 1.79D (range 0.5 – 5.5).

**Table 1:** Refractive Error in the Right Eyes of Examined Children (N,%).

		Age		
	3 Years (N = 6)	4 Years (N = 48)	5 Years (N = 25)	Total N = 79
Hyperopia (SE ≥ +0.50)	5 (83%)	33 (69%)	14 (56%)	52 (66%)
Emmetropia (-0.5 < SE < +0.5)	1 (17%)	12 (25%)	8 (32%)	21 (26%)
Myopia (SE ≤ -0.50)	0	3 (6%)	3 (12%)	6 (8%)
Astigmatism				
0.5-1.25	0	12 (25%)	6 (24%)	18 (23%)
1.5-3.75	1 (17%)	10 (21%)	4 (12%)	15 (19%)
4-5.75	0	0	2 (8%)	2 (2%)
Anisometropia				
Spherical (≥ 1.5)	0	0	1 (1%)	1 (1%)
Spherical (≥ 1.0)	0	3 (4%)	1 (1%)	4 (5%)
Cylindrical (≥ 1.5)	0	1 (1%)	0	1 (1%)
Cylindrical (≥ 1.0)	0	2 (3%)	0	2 (3%)
SE, spherical equivalent.				

Of those who underwent cycloplegia, 22 children (28%) were prescribed glasses for the first time and 16 children (20%) were found to have at least one refractive amblyopia risk factor (ARF). 11 children had only one refractive ARF, 9 were due to astigmatism alone and 2 were due to isolated hyperopia. The remaining 5 children had more than one refractive ARF, 4 had astigmatism combined with hyperopia and 1 child had astigmatism, hyperopia, and anisometropia. All ARFs discovered were due to visually significant refractive error as strabismus or media opacity was not seen in any children. Overall, there were 5 children with amblyopia, 4 bilateral and 1 unilateral, corresponding to 6% of the examined population and 3% of the screened population. All 4 cases of bilateral amblyopia were in children > 5 years of age. Of children with bilateral amblyopia, 3 had BCVA of 20/40 bilaterally and 1 had BCVA of 20/70 bilaterally. The cylinder error of all children with bilateral amblyopia was > 2.5D bilaterally, with a maximum of 5.5D. The spherical error of children with bilateral amblyopia varied, ranging from 0D to 4.5D.

Device screening results for examined children are presented in **Table 2**. Of the children who received cycloplegic examinations, the PlusoptiX referred 22 children (28%), 5 of which were classified as “unable.” The Retinomax referred 38 children (48%). 18 children were referred by both devices, 4 children were referred only by the PlusoptiX and 20 children referred only by the Retinomax. Of the 16 children found to have at least one refractive ARF, the PlusoptiX referred 15 (93.8%) and the Retinomax referred 16 (100%). The 5 children with amblyopia were referred by both devices. The average SE and spherical error in the right eyes of children referred by the Retinomax was 1.1D and 1.8D, respectively. The average SE and spherical error in the right eyes of children referred by the PlusoptiX was 1.6D and 2.7D, respectively. Of the 76 children who were screened but not examined, 24 (32%) were referred by the PlusoptiX and 44 (58%) referred by the Retinomax.

**Table 2:** Screening Results of Examined Children.

	Comprehensive Examination		
Screening Result	ARF present (+)	ARF absent (-)	Total
PlusoptiX S12C			
Refer	12	5	17
Pass	1	56	57
Inconclusive	3	2	5
Total	16	63	79
Retinomax K+3			
Refer	16	22	38
Pass	0	41	41
Inconclusive	0	0	0
Total	16	63	79

Screening for refractive ARFs, the PlusoptiX demonstrated an overall sensitivity, specificity, positive predictive value (PPV), and negative predictive value (NPV) of 93.8%, 88.9%, 68.2%, and 98.2%, respectively. The Retinomax had an overall sensitivity, specificity, PPV, and NPV of 100%, 65.1%, 42.1%, and 100%, respectively. Screening for the need for glasses, the sensitivity, specificity, PPV, and NPV of the PlusoptiX was 86.3%, 94.7%, 86.4%, and 94.7%, respectively. The sensitivity, specificity, PPV, and NPV of the Retinomax was 86.3%, 66.7%, 50%, and 92.7%, respectively.

## 4 Discussion

Programs and services that screen young children for visual impairment and disease such as refractive error, amblyopia, and strabismus are of great importance. The early detection and treatment of these conditions may lead to improved quality of life, better treatment outcomes, and may even prevent blindness. Automated screening instruments used by pediatricians and community programs have been shown to be efficient and effective tools for screening children for amblyopia and refractive error (9–11). The PlusoptiX S12 photoscreener and Retinomax K+3 autorefractor are two widely-used devices that have been validated for use in pediatric populations (12, 14–16).

This study evaluated the PlusoptiX S12 photoscreener and Retinomax K+3 autorefractor as vision screening devices used in a community vision screening program for preschool children. We screened 158 children with each device side-by-side at their respective preschools and provided comprehensive eye examinations with cycloplegia to 79 of those children. Of examined children, 28% were prescribed glasses for the first time and 20% were found to have at least one refractive ARF or amblyopia. The most common refractive ARFs detected during examination were due to astigmatism, hyperopia, or a combination of both. While both devices had a high sensitivity screening for refractive ARFs, we found the PlusoptiX performed better overall as a screening device for our program.

While various versions and models of the PlusoptiX and Retinomax have been compared to each other in previous studies (17–19), only one study has directly compared the same models evaluated in the current study (20). Kinori et al. compared the S12C and K+3 side-by-side and found the Retinomax performed better as a field screener for preschool children 3 to 5 years of age. Screening for the need for glasses, they found the Retinomax was 95% sensitive and 94% specific while the PlusoptiX was 86% sensitive and 84% specific (20). While they found the Retinomax superior in screening for the need for spectacle correction, our results differed as specificity, PPV, and NPV were all greater for the PlusoptiX compared to the Retinomax. Both devices had the same sensitivity of 86.3%.

The frequency of inconclusive screening results or “unables” by the PlusoptiX S12C reported by some studies has raised concerns about the utility of the device. Authors note that the significant number of “unables” may alter device specificity and PPV and lead to over referrals (20–22). Kinori et al. found that 70% of all referrals made by the PlusoptiX were due to “unables” and calculated a PPV of 43% screening for the need for glasses (20). Screening for ARFs in children aged 8.6-15.6 years, Crescioni et al. found inconclusive screening results constituted 46% of all S12C referrals with a device specificity of 61% (21). Using the S12C to determine the need for glasses in children aged 3 – 11 years in Turkey, Ugurbas et al. found “unables” accounted for 32% of referrals with a device PPV of 69% that increased to 83% when “unable” results were excluded from analysis (22).

Although high rates of inconclusive screenings have been noted to affect the usefulness of the S12C in some studies, this does not appear to be a consistent finding. In one study screening for ARFs in children aged 1-12 years, the sensitivity/specificity/inconclusive rate was 83%/86%/23% for the S12C (23). A 2021 study using a modified S12C to screen children for ARFs reported a sensitivity/specificity/inconclusive rate of 85%/96%/16% (24). Using the S12C to screen children with high prescreening prevalence of ARFs, Kirk et al. reported a sensitivity/specificity/inconclusive

rate of 91%/71%/10% (25). In the present study, there were 5 children who received cycloplegic examinations and were classified as “unable” by the PlusoptiX. Of those children, 3 had refractive ARFs, 2 had amblyopia, and all 5 received glasses. Our calculated sensitivity/specificity/inconclusive rate of 94%/89%/6% is consistent with the above studies and illustrates that inconclusive screenings do not necessarily lead to over referrals or lower device specificity or PPV. In contrast to the comparison of the S12C and K+3 by Kinori et al. (20), we found that the Retinomax had the potential to over refer, as it referred 78% more children with a lower specificity (65% vs 89%) and PPV (42% vs 68%) for identifying refractive ARFs compared to the PlusoptiX.

Studies have shown that non-cycloplegic screening with the Retinomax accurately detects refractive error (16, 26, 27). One study found that while the Retinomax has high sensitivity for detecting myopia, astigmatism, and anisometropia compared to cycloplegic retinoscopy, it may underestimate hyperopia when used without cycloplegia (18). In the present study, we found that the Retinomax had high sensitivity screening for refractive ARFs but had a low specificity of 65.1%. We found that the Retinomax referred 52% of screened children, a referral rate much higher than the 13% and 16% previously reported by other vision screening programs (11, 20). Given the higher referral rate and PPV of 42.1% screening for refractive ARFs, the Retinomax did not perform as well as the PlusoptiX as a screening device for our program. While the Retinomax was more sensitive and had a higher NPV than the PlusoptiX, it simply referred too many children to be considered the best choice for our program. One possible explanation for the difference in performance is the different referral criteria for each device. Referral criteria for the Retinomax were based on data from the VIP study, corresponding to a specificity of 90%; however, we did lower the threshold for anisometropia from ≥2.75D to ≥1.50D in an effort to increase device sensitivity (28, 29). Referral criteria for the PlusoptiX were also based on a specificity of 90%, set by the manufacturer. It is possible that a modification of the device referral criteria may improve the specificity of the Retinomax, although additional studies are warranted to explore this further.

There are a few limitations to the study. The participating children were all attending preschools from one school district in Southern California. Also, all children were enrolled in Head Start, a program for low-income families. While we did not collect demographic data, the study took place in a majority Hispanic/Latino school district (30). Thus, the results from this study may not apply to individuals of different ages, geographic regions, or economic status. Additionally, one screener used the PlusoptiX and another screener used the Retinomax for all screenings. While both screeners were trained with the devices and had experience screening preschool aged children prior to the study, variations in screening technique between individual screeners could affect the amount of “unable” screening results reported by the PlusoptiX and may have influenced our results. Another limitation to the study is that refractive error measurements taken by both devices on initial screening were not recorded. As only the screening result of “pass” or “refer” for each child was recorded, we are unable to compare refractive error measurements obtained by the devices to cycloplegic retinoscopy measurements. Such analysis would have been valuable for exploring various factors that potentially led to the difference in referral rates between the devices. Therefore, our comparison and analysis of the devices is incomplete, and our results should be taken judiciously. Finally, we were only able to provide cycloplegic examinations to 79 children, approximately half of all children screened. Due to this lower sample size, the prevalence of refractive error, amblyopia, and refractive ARFs reported here may not accurately represent our screened population. However, we believe that the examined population is representative of the screened population, as the device referral rates between each group were similar. In the examined group, referral rates for the PlusoptiX and Retinomax were 28% and 48%, respectively, while in the unexamined group, referral rates were 32% and 58%, respectively. Still, we would have ideally examined all children who were screened, and further research exploring the barriers to pediatric vision care is needed.

Overall, our study showed that the Retinomax was more sensitive, had a higher NPV, and referred more children while the PlusoptiX was more specific and had a higher PPV in screening children for refractive ARFs. Based on the data from our study, we believe that the PlusoptiX performed better overall as a screening device for our program, as the Retinomax referred too many children to be feasible.

## Data availability statement

The original contributions presented in the study are included in the article/supplementary material. Further inquiries can be directed to the corresponding author.

## Author contributions

SH, DS, IM, and JE designed the study. SH, IM, and JE executed the study and acquired subject data. SH analyzed the data and wrote the manuscript. SH and DS reviewed and revised the manuscript. All authors contributed to the article and approved the submitted version.
