# Corrigendum: Automated screening devices for vision screening in preschool children: a comparison of the PlusoptiX S12C photoscreener and retinomax K+3 autorefractor

**DOI:** 10.3389/fopht.2023.1215403

**Published:** 2023-07-06

**Authors:** Stephen C. Hunter, Donny W. Suh, Iliana Molina, Jennifer Espinoza

**Affiliations:** ^1^ University of California Riverside School of Medicine, Riverside, CA, United States; ^2^ Department of Ophthalmology, Gavin Herbert Eye Institute, University of California Irvine School of Medicine, Irvine, CA, United States

**Keywords:** vision screening, children, amblyopia, refractive error, amblyopia risk factor, photoscreener, autorefractor

In the published article, there was an error in the author list, and authors Iliana Molina and Jennifer Espinoza were erroneously excluded. The corrected author list appears below.

Stephen C. Hunter^1*^, Donny W. Suh^2^, Iliana Molina^2^ and Jennifer Espinoza^2^

^1^ University of California Riverside School of Medicine, Riverside, CA, United States

^2^ Department of Ophthalmology, Gavin Herbert Eye Institute, University of California Irvine School of Medicine, Irvine, CA, United States

The authors apologize for this error and state that this does not change the scientific conclusions of the article in any way. The original article has been updated.

In the published article, there was an error. The Author Contributions and Acknowledgements sections contained incorrect information and have been updated to reflect the recognition of Iliana Molina and Jennifer Espinoza as authors.

A correction has been made to the Acknowledgements section. The section previously read:

“The authors wish to thank Iliana Molina and Jennifer Espinoza for their assistance with data acquisition, data processing, and for their contributions to the UCI EyeMobile for Children program”.

The corrected sentence appears below:

“The authors wish to thank the UCI EyeMobile for Children program for providing valuable vision screening services to children and supporting this research”.

A correction has been made to the Author Contributions section. The section previously read:

“SH and DS designed the study. SH analyzed the data and wrote the manuscript. SH and DS reviewed and revised the manuscript. All authors contributed to the article and approved the submitted version”.

The corrected sentence appears below:

“SH, DS, IM, and JE designed the study. SH, IM, and JE executed the study and acquired subject data. SH analyzed the data and wrote the manuscript. SH and DS reviewed and revised the manuscript. All authors contributed to the article and approved the submitted version”.

The authors apologize for this error and state that this does not change the scientific conclusions of the article in any way. The original article has been updated.

